# Antioxidant PLA Composites Containing Lignin for 3D Printing Applications: A Potential Material for Healthcare Applications

**DOI:** 10.3390/pharmaceutics11040165

**Published:** 2019-04-04

**Authors:** Juan Domínguez-Robles, Niamh K. Martin, Mun Leon Fong, Sarah A. Stewart, Nicola J. Irwin, María Isabel Rial-Hermida, Ryan F. Donnelly, Eneko Larrañeta

**Affiliations:** School of Pharmacy, Queen’s University Belfast, 97 Lisburn Road, Belfast BT9 7BL, UK; j.dominguezrobles@qub.ac.uk (J.D.-R.); nmartin24@qub.ac.uk (N.K.M.); mfong03@qub.ac.uk (M.L.F.) sstewart35@qub.ac.uk (S.A.S.); n.irwin@qub.ac.uk (N.J.I.); mariaisabel.rial@usc.es (M.I.R.-H.); r.donnelly@qub.ac.uk (R.F.D.)

**Keywords:** 3D printing, fused filament fabrication, lignin, antioxidant materials, wound dressing

## Abstract

Lignin (LIG) is a natural biopolymer with well-known antioxidant capabilities. Accordingly, in the present work, a method to combine LIG with poly(lactic acid) (PLA) for fused filament fabrication applications (FFF) is proposed. For this purpose, PLA pellets were successfully coated with LIG powder and a biocompatible oil (castor oil). The resulting pellets were placed into an extruder at 200 °C. The resulting PLA filaments contained LIG loadings ranging from 0% to 3% (*w*/*w*). The obtained filaments were successfully used for FFF applications. The LIG content affected the mechanical and surface properties of the overall material. The inclusion of LIG yielded materials with lower resistance to fracture and higher wettabilities. Moreover, the resulting 3D printed materials showed antioxidant capabilities. By using the 2,2-diphenyl-1-picrylhydrazyl (DPPH) method, the materials were capable of reducing the concentration of this compound up to ca. 80% in 5 h. This radical scavenging activity could be potentially beneficial for healthcare applications, especially for wound care. Accordingly, PLA/LIG were used to design meshes with different designs for wound dressing purposes. A wound healing model compound, curcumin (CUR), was applied in the surface of the mesh and its diffusion was studied. It was observed that the dimensions of the meshes affected the permeation rate of CUR. Accordingly, the design of the mesh could be modified according to the patient’s needs.

## 1. Introduction

The interest in additive manufacturing (commonly known as 3D printing) for biomedical applications has increased significantly during the last decade [1]. Among all the different types of 3D printing, fused filament fabrication (FFF) is the most commonly used [2]. In FFF, a polymer filament is heated and extruded through a small nozzle and subsequently solidified on a build plate [3]. FFF gained popularity quickly after the RepRap project in 2005 [4]. This project was focused on developing low cost do-it yourself FFF printers [4]. Accordingly, different biomedical and pharmaceutical applications of additive manufacturing have been described during the last decade [1,5,6]. These applications include drug delivery systems, prosthetics or implantable devices [1,5,6,7]. The most common material used for FFF is poly(lactic acid) (PLA). This is due to its processability for extrusion applications, high mechanical strength and low coefficient of thermal expansion [8]. Moreover, PLA is a renewable, biodegradable and biocompatible polymer [7,9]. These properties make this material ideal for pharmaceutical and biomedical applications.

To provide extra features to PLA, different strategies have been followed. The combination of PLA with other molecules that can give added value to the final composite material for healthcare applications have been explored in the past [10]. One of the potential scenarios is the incorporation of molecules with antioxidant properties to PLA. The unrestrained production of free radicals and reactive oxygen species is linked with the onset of diseases such as rheumatoid arthritis, atherosclerosis or cancer [11]. Accordingly, the development of antioxidant compounds/materials can contribute to reduce the concentration of these compounds [12]. Moreover, it has been shown that the excess of reactive oxygen species prevents wound healing [13,14]. Accordingly, antioxidants have been proposed as a way to control oxidative stress in wounds to accelerate their healing.

The use of antioxidant compounds for 3D printing applications has not been extensively explored in the past. Van Lith et al. proposed the use of stereolithography (an alternative to FFF 3D printing technology) to create bioresorbable antioxidant vascular stents [15]. The stents provided antioxidant capabilities that are beneficial to reduce oxidative stress Another application of antioxidant compounds for 3D printing applications was described by Lücking et al. [16]. In this work, another type of 3D printing, selective laser sintering, was used to prepare materials containing antioxidant to enhance cell proliferation [16]. They used UV-stable polyamide 12 material with antioxidant properties. However, the nature of the antioxidant compound was not disclosed. 

An interesting renewable and natural compound with antioxidant properties is lignin (LIG). LIG is a biopolymer present in the support tissues of vascular plants and provides chemical and mechanical protection from external stresses [17]. Moreover, it has been reported that LIG presents antioxidant and antimicrobial properties [18,19,20,21]. Among other alternatives, LIG-based materials have attracted the attention of researchers [17,22,23,24,25,26,27]. Considering that LIG is the second most abundant polymer on Earth [17,24], the applications of LIG to develop new materials are unexploited as only a 2% of the total LIG production (ca. 70 million tons) is reused for specialty products [17]. The rest is used as burning fuel or treated as a waste [17,28]. Due to its high availability and its antimicrobial and antioxidant properties, LIG has potential for biomedical applications. These are not the only advantages as LIG is a relatively cheap to obtain with prices ca. €33 per ton [29]. To date, the biomedical applications of this biopolymer, remain relatively unexplored. A few scientific articles can be found describing its use to prepare coatings, nanoparticles or hydrogels [21,30,31,32] for potential medical applications. 

In the present work, we propose the combination of LIG with PLA to create filament that can be used for healthcare FFF applications. These compounds were combined via hot melt extrusion. The resulting composite materials were characterized and successfully used for fused deposition modeling applications. This approach has potential for multiple applications but in the present study we hypothesized that it can be used for wound dressing applications. The antioxidant properties of the material could potentially enhance wound healing. The resulting polymeric composite material has antioxidant properties and do not rely on the release of an antioxidant agents. Accordingly, the material can provide localized and prolonged antioxidant activity. Finally, the proposed method is simple and allows combination of PLA with LIG and additional molecules such as antibiotics. In this way, the resulting material can have advanced properties. For the present work we selected tetracycline (TC) as in this way the material can be used to prevent wound infections.

## 2. Materials and Methods 

### 2.1. Materials

Ingeo PLA 3D850 biopolymer (PLA pellets) was purchased from NatureWorks LCC (Minnetonka, MN, USA). This PLA grade (3D850) has been specifically developed for FFF applications, having an excellent processability and printability. 

LIG sample (BioPiva 100) was a softwood Kraft LIG acquired from UPM (Helsinki, Finland). This LIG sample was used as provided (63–68% dry matter content). However, the higher moisture content of this sample was considered for any following calculations. An important part of the characterization of this aromatic polymer was kindly provided from the supplier. Klason LIG content (TAPPI T 222 om-02) was around 92% of the dry matter and acid-soluble LIG (TAPPI UM 250) was around 4% of the dry matter. On the other hand, the total amount of carbohydrates (SCAN-CM 71:09) accounted for around 2% of the dry matter and the content of inorganic particles (internal method, 700 °C) accounted for around 1% of the dry matter. Molar mass of this LIG sample was 5000—6000 Da. Finally, the particle size (median d_50_) was around 20 µm and it was analyzed using a Mastersizer 3000 (Malvern, UK). 

Castor oil used to attach LIG and TC to the pellets surface was purchased from Ransom Naturals Ltd. (England, UK). TC was acquired from Honeywell Fluka™ (Leicestershire, UK). To perform the release study, Hydroxypropyl-methyl cellulose (HPMC) was acquired by Colorcon Limited (Dartford, UK), ascorbic acid (AA) was provided by DSM (Heerlen, the Netherlands), tween 80 (T80) was obtained from Tokyo Chemical Industry UK Ltd. (Oxford, UK) and curcumin (CUR), the model drug used in this study, was purchased from Cayman Chemical (Michigan, USA). Finally, poly(vinyl alcohol) (PVA) filament was obtained from Ultimaker B.V., The Netherlands (Diameter: 2.85 mm; Melting Temperature: 163 °C).

*Staphylococcus aureus* (ATCC 6538; LGC Standards, Middlesex, UK) were maintained on cryopreservative beads in 10% glycerol at −80 °C and cultivated in Mueller Hinton broth (MHB) at 37 °C when required for the microbiological assessments.

### 2.2. LIG-PLA Pellets Production

The method used to produce these LIG-PLA coated pellets was previously described by Weisman et al. [10]. Briefly, 50 mL Falcon tube was filled with PLA pellets (40 g). Then, castor oil (40 µL) was added into the tube and it was vortexed until the pellets were properly coated. Subsequently, these castor oil covered pellets were placed into a new 50 mL Falcon tube and the LIG or LIG and tetracycline powder were added, and it was vortexed again. Different batches containing 0.5%, 1%, 2% and 3% (*w*/*w*) of LIG coating and 1% and 2% (*w*/*w*) of LIG and TC, respectively, were prepared. Additionally, castor oil covered PLA pellets and only PLA pellets were used as control samples to make the filaments. 

### 2.3. Extrusion of Filaments

The Next 1.0 filament extruder (3devo, Utrecht, The Netherlands) was used to prepare the different filaments. The temperature was adjusted through a control panel positioned at the side of the extruder and it was between 170 and 190 °C, due to the existence of 4 heaters. Moreover, the extruder speed was established at 5.0 rpm and the fan speed was 70%. Additionally, 2.85 mm was selected as the diameter of the extruded filaments.

### 2.4. Fused Filament Fabrication

Once the filaments were extruded, discs and squares were 3D-printed using an Ultimaker 3 (Ultimaker B.V., Geldermalsen, The Netherlands) FFF system and Cura^®^ software between 185 and 205 °C. The Ultimaker 3 FFF system was equipped with two extruders containing a 0.4 mm nozzle. It is important to note that this equipment is a RepRap Open Source FFF equipment [4]. Thermogravimetric analyses were performed to ascertain the stability of LIG at the process temperatures (data not shown), showing that a small amount of LIG was degraded during the process (ca. 3%). 

### 2.5. Material Characterization

#### 2.5.1. Microscopy

The morphologies of the materials prepared using FFF as well as the filaments and pellets were assessed using a Leica EZ4 D digital microscope (Leica, Wetzlar, Germany).

#### 2.5.2. Contact Angle Measurement

The influence of the LIG on the contact angle of water with the surface of the 3D printed materials (squares) was assessed using an Attension Theta equipment (Attension Theta, Biolin Scientific, Gothenburg, Sweden). OneAttension software analyzed results to give an indication of the wettability of the surface. Measurements were performed in triplicate. 

#### 2.5.3. Thermal Properties

The glass transition temperature (*T_g_*) of the 3D printed materials as well as the pure materials (PLA, LIG, tetracycline and castor oil) was measured using DSC Q100 differential scanning calorimeter (TA instruments, Bellingham, WA, USA). Scans were run from 30 to 300 °C at 10 °C/min under a nitrogen flow rate of 10 mL/min. 

#### 2.5.4. Stability Study

For the degradation experiments, the 3D printed discs were incubated at 37 °C in screw-capped vials containing 3 mL of phosphate-buffered saline (PBS) (pH = 7.4) over a period of 30 days. The specimens incubated were run in triplicate. After different interval times, the discs were separated from the degradation medium and the excess of it was removed with a tissue paper and subsequently they were dried at 80 °C in an oven for 5 min. The degradation medium or PBS was replaced after each time interval and the mass loss was measured. 

#### 2.5.5. Mechanical Properties

The break strength of the different filaments extruded were evaluated using a TA.XTplus texture analyzer (Stable Micro Systems, Surrey, UK) in compression mode. Pieces of filaments of 2 cm were cut and used for the test. Then, these samples were placed on two aluminum blocks (with 1 cm of separation) and a tapered aluminum probe (5.5 cm in length with a blunt end of radius 1.0 mm) was moved towards the pieces of filaments. The probe moved at a speed of 2 mm·s^−1^ with a maximum distance of travel of 5 mm. The filament failure force was assumed to be the peak maximum of the force-distance curve.

### 2.6. PLA-LIG Mesh Production and CUR Release for Potential Wound Healing Applications

**Figure 1:**
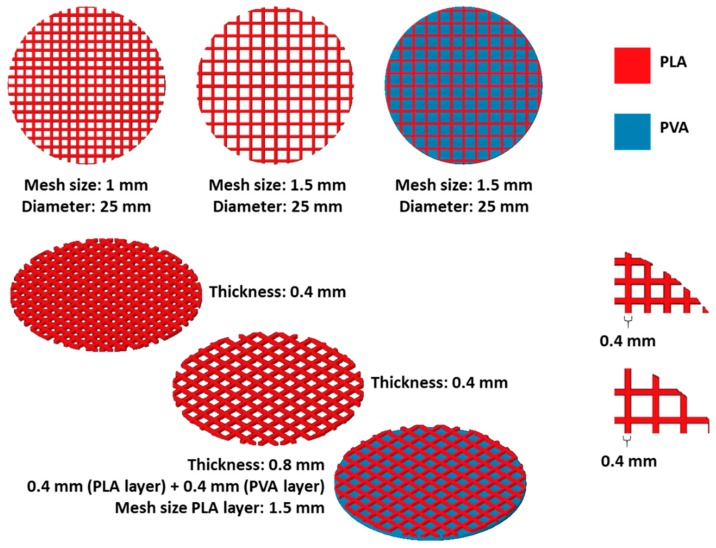
Scheme of the different meshes produced using FFF.

Different 3D printed meshes (0.4 mm thickness) made using the filament containing 2% of LIG were used for CUR permeation experiments. Figure 1 shows the different types of meshes that were prepared and their dimensions. Moreover, combined meshes were prepared using a two layered distribution (0.4 mm each layer). The first layer contained a PLA/LIG mesh and the second layer was printed using PVA. For these purpose, horizontal diffusion cell system was used with some modifications. Each side of the system was employed as an individual replicate for the different mesh systems. Curcumin was used as the model drug for this test. Films were prepared with HPMC and CUR. For his purpose, a solution containing 10% (*w*/*w*) of HPMC and an excess of CUR was prepared in ethanol-water (70% ethanol). The solution was centrifuged to remove the non-dissolved CUR. Subsequently, films were casted using this solution. Then, a 1 cm diameter cork-borer was employed to cut this film into discs, which were placed together the different 3D printed meshes in the cells. Then, each cell of the system was filled with 3 mL of a solution containing 10% (*w*/*v*) of Tween 80 and 1 mg/mL of ascorbic acid prepared in PBS, which was stirred and thermostatically maintained at 37 ± 1 °C. Samples (≤0.5 mL) were removed from the sampling arms of the cells at predetermined time intervals and replace with fresh release media. The concentration of CUR was evaluated using a UV-visible plate reader (PowerWave XS Microplate Spectrophotometer, Bio-Tek, Winooski, VT, USA) at a wavelength of 425 nm.

### 2.7. Antioxidant Activity

DPPH (2,2-diphenyl-1-picrylhydrozyl) radical was employed to measure the antioxidant activity of 3D printed materials based on the radical scavenging property of the LIG [33]. Briefly, 3 mL of a DPPH solution dissolved in methanol (23.6 mg/L) was added to the 3D printed samples (a square of 1 cm × 1 cm × 0.1 cm) placed in a 24-well plate. A control sample of 23.6 mg/L of DPPH in methanol was also measured. The samples were then incubated in the dark for 300 min at room temperature. At predetermined time intervals (each 60 min), 300 µL samples were collected and the well was immediately replenished with an equivalent volume of methanol. The absorbance of the different solutions was measured at 517 nm in triplicate using a UV–vis plate reader (PowerWave XS Microplate Spectrophotometer, Bio-Tek, Winooski, VT, USA). The residual DPPH content in the solution was calculated using Equation (1)

Residual DPPH content (%) = 100 − 100 (*A*_0_ − *A*_1_/*A*_0_) (1)

where *A*_0_ is the absorbance of the control sample and *A*_1_ is the absorbance in the presence of the sample at any time. Decreased absorbance of the reaction indicates a stronger DPPH radical scavenging activity.

### 2.8. Antimicrobial Properties

The in vitro microbiological analysis was performed according to the previous published works [34,35]. In brief, a bacterial suspension of *S. aureus* (1 × 10^8^ cfu mL^−1^) in PBS and supplemented with 0.5% TSB (pH 7), was diluted (1:100) with PBS containing 0.5% TSB. Replicate samples of 3D printed squares (1 cm × 1 cm × 0.1 cm) were placed in individual wells of a 24-well plate and then aliquots of 1 mL of the diluted bacterial suspension with a density of 1 × 10^6^ cfu mL^−1^ was added completely covering the 3D printed squares. The plate was continuously shaken in an orbital incubator at 37 °C for 24 h. Then the samples were removed from the 24-well plate containing the bacterial suspension and the non-adherent bacteria were removed by several washing steps, first in PBS (1 × 10 mL), and then in quarter-strength Ringer’s solution (QSRS) (3 × 10 mL) [36]. After the wash step, 3D printed squares were transferred into fresh QSRS (5 mL), sonicated (15 min) and vortexed (30 s) to remove adherent bacteria. The sonication technique has previously been demonstrated not to affect bacterial viability or morphology [37]. A viable count of the QSRS was performed by the Miles and Misra serial dilution technique [38] followed by plating onto Mueller–Hinton agar to enumerate the previously adhered bacteria per sample. 

### 2.9. Statistical Analysis

All data were expressed as mean ± standard deviation. Data were compared using a one-way analysis of variance (ANOVA), with Tukey’s HSD post-hoc test. In all cases, *p* < 0.05 was the minimum value considered acceptable for rejection of the null hypothesis.

## 3. Results

### 3.1. PLA and LIG Composite Material Preparation and Characterization

The antioxidant capabilities of LIG have been reported multiple times in the past. Considering the potential health benefits associated with antioxidant materials, LIG has potential to be combined with 3D printable biocompatible polymers for biomedical applications. For this purpose, PLA was selected as the ideal material to be combined with LIG. It has been extensively used for FFF 3D printing applications and it is biocompatible and biodegradable [7,9]. 

**Figure 2:**
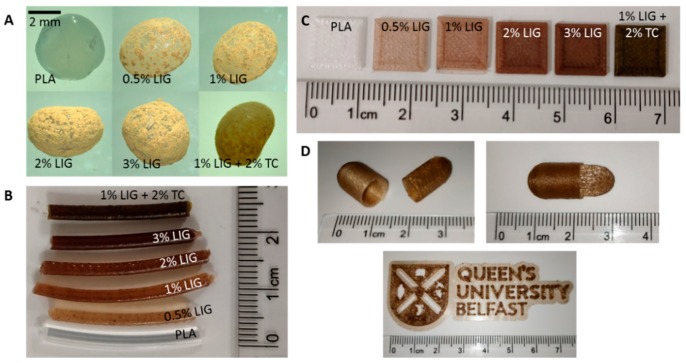
Photographs of: PLA and PLA coated pellets (**A**); LIG and TC containing PLA filaments (**B**); LIG and TC containing 1 cm × 1 cm squares prepared using 3D printing (**C**); and different shapes printed using the filament containing 2% (*w*/*w*) LIG (**D**).

Hot melt extrusion was used to combine LIG and PLA to form a composite material. PLA was supplied in pellet form while LIG was supplied in powder form. To get a homogeneous mixture of both types of materials, a coating approach was used. Castor oil was used to coat the PLA pellets and, subsequently, LIG powder was added to the mixture. This method was described before to combine PLA with chemotherapeutic and antibiotic drugs. Figure 2A shows the image of the LIG coated pellets containing LIG concentrations ranging from 0.5% to 3% (*w*/*w*). When higher amounts of LIG were added, the pellets showed a higher coating degree. TC, an antibiotic compound with reported antioxidant capabilities, was combined with PLA and LIG. These pellets contained a 2% (*w*/*w*) of TC and 1% (*w*/*w*) of LIG. The coating of these pellets was homogeneous, as it can be seen in Figure 2A. Finally, 3% was the maximum LIG loading that was evaluated, as, in this case, the pellets were completely coated by LIG powder (Figure 2A).

PLA was combined with LIG and TC successfully using hot melt extrusion. Figure 2B shows fragments of the obtained filaments. Moreover, the filaments were successfully used for FFF application, as sshown in Figure 2C. Squares (1 cm × 1 cm) were successfully printed using the PLA/LIG composites. However, the filaments could be used successfully to prepare more complex geometries as it is illustrated in Figure 2D.

The proposed method is a good alternative to combine PLA and LIG, as it provided a good mixture of both components and did not require the use of any solvents. In the past, LIG and PLA have been combined using a casting method [39]. This method requires the use of solvents that could present toxicity issues for healthcare applications. 

**Figure 3:**
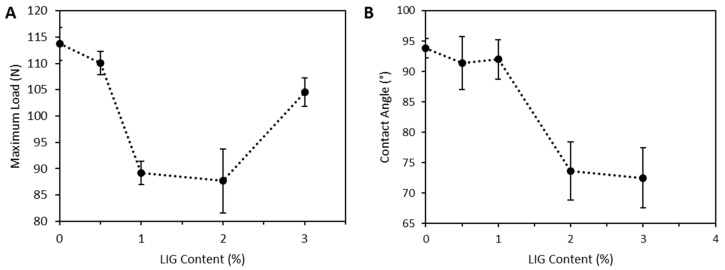
Maximum load before fracture for LIG containing filaments (*n* = 5) (**A**); and contact angle of water with the surface of 3D printed materials obtained using PLA/LIG composites (*n* = 4) (**B**).

LIG/PLA filaments showed lower resistance to fracture than PLA filaments. Figure 3A shows that the presence of LIG in the material reduced the maximum load that the materials can resist before fracture. There were no significant differences between the maximum load obtained for materials containing 0% and 0.5% (*w*/*w*) of LIG (*p* = 0.506). Interestingly, the maximum load dropped when the LIG content was increased from 0.5% to 1% (*w*/*w*) (*p* < 0.05). Moreover, when the LIG content reached 3%, the resulting materials showed higher resistance to fracture (*p* < 0.05).

The surface properties of the 3D printed materials were evaluated. Figure 3B shows the contact angle of water with the material surface. Interestingly, there were no significant differences between the contact angles obtained for PLA + castor oil and the materials containing up to 1% of LIG (*p* > 0.05). Castor oil did not influence in the contact angle of the materials. The obtained contact angles for PLA and PLA/castor oil showed no significant differences between them (*p* = 0.995). However, the materials showed a noticeable reduction in their wettability when the LIG content was increased up to 2% (*w*/*w*). The measured contact angle between materials containing 2% and 3% of LIG were significantly lower than the previously described materials (*p* < 0.05). Additionally, the obtained contact angles for these materials showed similar values.

**Figure 4:**
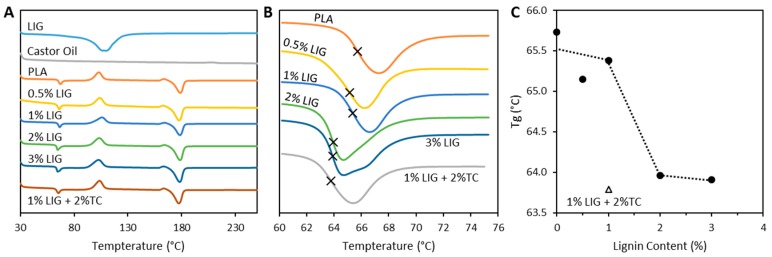
DSC thermograms obtained for LIG, PLA, castor oil and the resulting PLA/LIG and TC composites (**A**); expanded view of the thermogram between 60 and 76 °C (**B**); and *T_g_* variation as a function of the LIG content (**C**).

The materials were analyzed using FTIR spectroscopy (data not shown). No differences were observed in the spectra of pure PLA and LIG containing materials. This is due to the lower LIG loading within the materials. DSC measurements were performed to evaluate the interaction between LIG and PLA. Figure 4A shows the DSC thermograms of LIG, PLA, castor oil and the 3D printed PLA/LIG composites. This figure shows that PLA/LIG composites showed the same transitions that can be observed for PLA. The first transition observed was a glass transition (*T_g_*) at ca. 65 °C. PLA melting point was observed at ca. 180 °C. When LIG was incorporated into the material, a reduction in the *T_g_* was observed (Figure 4B,C). The materials containing 0.5% and 1% of LIG showed almost the same *T_g_* value as pure PLA. However, when LIG loading increased up to 2%, a *T_g_* reduction was observed (Figure 4B,C). Composite materials containing 3% of LIG showed similar behavior. Interestingly, the melting temperature of the materials was not affected by the LIG presence. Finally, the stability studies showed that the materials did not lose weight after 30 days in PBS (*p* < 0.05) (data not shown).

### 3.2. PLA and LIG Composite Antioxidant and Antimicrobial Properties

**Figure 5:**
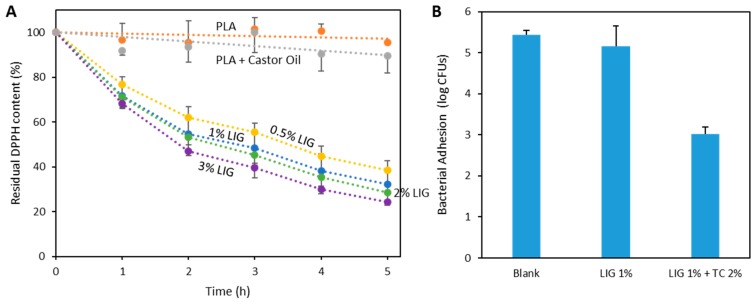
Residual DPPH content as a function of time for the LIG containing composites (*n* = 3) (**A**); and bacterial adhesion to the PLA/LIG and PLA/LIG/TC composites (*n* = 3) (**B**).

Figure 5A shows the radical scavenging activity of PLA/LIG 3D printed composites. The presence of LIG in the material gave the material antioxidant properties. PLA and PLA + castor oil based materials did not show a DPPH concentration reduction over time. Moreover, materials containing LIG showed higher radical scavenging activity as the presence of DPPH decreased over time. This indicates that the presence of LIG provides the antioxidant activity to the materials. As expected, materials with higher LIG content showed higher antioxidant activity as they were more efficient in reducing the DPPH concentration over time. 

It has been reported previously that LIG has antimicrobial activity. Accordingly, the antimicrobial capabilities of the 3D printed materials were evaluated by studying bacterial adhesion. The adhesion of *S. aureus* to the material was evaluated (Figure 5B). When comparing the bacterial adhesion of PLA/castor oil (blank) and the 3D printed material containing 1% (*w*/*w*) LIG, there were no significant differences (*p* = 0.980). Accordingly, it can be established that the presence of 1% (*w*/*w*) LIG did not provide any antimicrobial capabilities to the material. Moreover, similar results were obtained for higher concentrations of LIG (*p* > 0.05) (data not shown). Accordingly, we can establish that the selected concentrations of LIG were not adding any antimicrobial capabilities to the composite materials. However, the addition of the TC showed significant reductions in bacterial adherence (Figure 5B) (*p* = 0.001). TC is an antibiotic compound and accordingly can reduce the bacterial load attached to the surface of the material.

### 3.3. 3D Printed PLA and LIG Composite Meshes for Potential Wound Healing Applications

**Figure 6:**
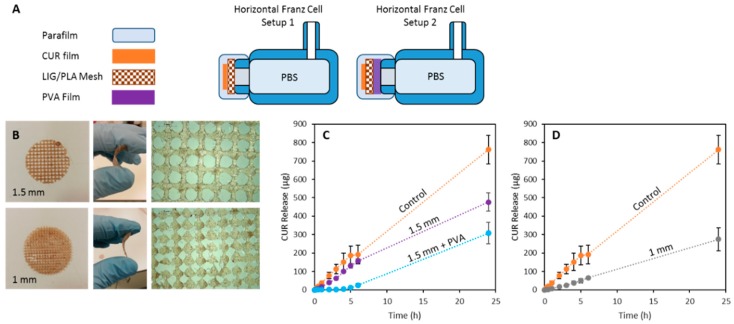
Experimental setup used to measure drug diffusion trough the 3D printed meshes (**A**); photographs of the 3D printed meshes made of PLA and 2% (*w*/*w*) LIG (**B**); and CUR release through 1.5 mm (**C**) and 1 mm (**D**) 3D printed meshes (*n* = 3).

As described previously, antioxidant materials can be extremely beneficial for healthcare applications such as wound healing. Accordingly, the proposed materials can be easily integrated in wound dressings. For this purpose, 3D printed meshes were prepared using PLA/LIG composite materials. These meshes can provide mechanical protection to the wound while providing antioxidant activity. Due to their design, soluble patches containing drugs can be applied to the surface of the mesh. The drug can diffuse through the mesh pores to the wound. An experimental setup was prepared to evaluate the delivery of an antioxidant and wound healing model compound, CUR (Figure 6A). Two different types of meshes were prepared containing two different grid sizes (1 and 1.5 mm) (Figure 6B). As shown in Figure 6B, the materials were flexible and thus could adapt to the surface of the wound.

Figure 6C shows the permeation of CUR through the meshes. The 1.5 mm meshes provided a slower CUR release than the control (CUR containing film alone). This effect was more substantial when the mesh size was reduced to 1 mm (Figure 6D). Moreover, the release rate could be delayed by combining the mesh with a soluble PVA film. The PVA film could be printed in combination with the LIG/PLA mesh using fuse deposition modeling equipment equipped with a dual extruder. Figure 6C shows how the incorporation of a PVA film with a LIG/PLA mesh delayed CUR release.

## 4. Discussion

Human population growth and industrial development are generating an increasing demand for polymeric materials. The majority of these types of materials are derived from the petrochemical industry and, accordingly, they have an enormous impact on the environment [40]. Therefore, the scientific community is developing green and sustainable alternatives to the traditional polymeric materials [17]. As mentioned previously, LIG has potential to be used as a functional additive due to its interesting properties.

The antioxidant properties of LIG can be used for potential healthcare related applications such as wound healing. Reactive oxygen species are strongly linked to the pathogenesis of chronic wounds [41]. Accordingly, as antioxidant materials contribute to reduce the concentration of these species, they have potential to be applied as wound healing materials. Moreover, it has been shown that LIG nanofibrous dressings contribute to wound healing [42].

PLA is a biocompatible and biodegradable material. Accordingly, by combining it with LIG materials, added value can be obtained. Due to the flexibility of PLA for 3D printing applications, the resulting composite materials can be produced in any shape. This allows physicians to prepare dressing for patients on demand modifying the size and shape. Finally, due to the biodegradable nature of PLA, the combination of PLA and LIG yields green materials.

The present work showed that PLA and LIG can be combined easily by coating PLA pellets with LIG. Other alternatives to prepare PLA/LIG composites have been explored but they require organic solvents or more complex equipment such as twin screws extruders [39,43,44].

Ye et al. described a method to prepare LIG/PLA composites by using a casting method. In this work, they achieved higher LIG loadings [39]. The composite materials described in that work showed similar properties to those described in this paper. Their PLA-based composites showed a contact angle reduction for the composites containing higher LIG loadings [39]. In the present work, the 3D printed composites containing 3% (*w*/*w*) and 2% (*w*/*w*) of LIG showed contact angles of ca. 75° (Figure 3B). In contrast, Ye et al. reported loadings of up to 20% (*w*/*w*) to obtain a similar effect. Accordingly, LIG improve the wettability of the materials. Moreover, Ye et al. did not report any *T_g_* changes for the composites. In the present work, materials containing LIG contents ≥2% (*w*/*w*) showed a *T_g_* reduction (Figure 4). Accordingly, it can be established that small amounts of LIG can contribute to change PLA chain mobility within the glass transition region [43]. Similar behavior was observed by Kai et al. [45].

Moreover, the mechanical properties of the resulting material showed that the produced LIG containing filaments showed lower resistance to load than pure PLA. However, this effect was not obvious for composites containing 3% (*w*/*w*) of LIG. In this case, the materials showed an increase in the maximum load before fracture. 

The antioxidant properties of LIG-containing 3D printed materials were evaluated by using the DPPH assay (Figure 5A). Similarly, Kai et al. investigated the antioxidant properties of PLA and LIG nanofibers [45]. They obtained DPPH concentration reductions up to ca. 60% after 72 h [45]. However, in the present study, after 5 h, the DPPH concentration reduction was ca. 80%. Moreover, these nanofibers contained up to 50% (*w*/*w*) LIG. Accordingly, the materials described in the present papers showed a more efficient antioxidant capability. There are a wide variety of antioxidant compounds described in the literature, such as resveratrol or curcumin. However, these compounds are generally expensive. Considering that LIG can be considered a reduced cost additive [29], the combination of PLA with LIG is a more viable option to obtain antioxidant 3D printable composites. Finally, there are a wide variety of wood-based filaments for FFF. These products are made using PLA, ABS or similar polymers combined with wood particles or sawdust [46]. The antioxidant compounds from wood need to be isolated before they can have its effect [47]. Therefore, the direct combination of LIG with PLA seems to be the best approach to obtain low-cost green 3D printable biomaterials with antioxidant properties.

The present method used to obtain LIG containing filaments for 3D printing application can be used to incorporate multiple compounds. It was demonstrated that an antibiotic, TC, can be incorporated into the filaments (Figure 2). Accordingly, the materials containing 2% (*w*/*w*) of TC showed effective reduction of *S. aureus* adhesion to the materials (Figure 5B). Moreover, LIG did not show any antibacterial activity in this case. It has been reported in the past that LIG can be added to materials to provide antibacterial activity against Gram-positive (*S. aureus*) and Gram-negative (*P. mirabilis*) bacteria [21]. However, the present study showed that LIG did not provide antibacterial activity at the selected concentrations. Accordingly, LIG and TC can be combined effectively with PLA to provide antioxidant and antimicrobial activities. This combination of molecules is extremely beneficial for wound dressings for chronic wounds as the materials can contribute to wound healing and to prevent infections.

Finally, the selected materials can be easily shaped as meshes (Figure 6B). These meshes can be adapted to the wound site due to their flexibility while providing some degree of protection. Moreover, the mesh design can be used to administer therapeutic compounds through the pores to the wound surface. In the present work, we used CUR as a model molecule. CUR has been extensively used for wound healing purposes [48]. Accordingly, films containing CUR were prepared and its permeation through the meshes was studied (Figure 6C,D). As expected, the permeation of CUR through the meshes was slower than the dissolution of a disc containing CUR alone. Moreover, permeation through the 1.5 mm meshes was quicker than the permeation through 1 mm ones. By controlling the geometry, the permeation rate can be controlled. Accordingly, this can be adapted to the needs of the patients. 

Modern FFF systems contain a two-nozzle configuration that can be used to prepare devices containing two different materials. In the present work, we designed a combined patch that contained a PVA film in contact with the release medium. In this way, a delayed release was obtained (Figure 6C). This PVA film could play a double role, providing a moist environment to the wound while delaying and controlling the CUR release. It has been reported previously that a moist wound environment improves the wound closure while reducing pain and scar formation [49].

Overall, the present paper describes a simple method to combine an antioxidant renewable compound, LIG, with PLA for 3D printing applications. A potential scenario for this material is as a wound dressing material due to the antioxidant activity of the composite material that can contribute to wound closure. Due to the low price of 3D printing equipment and its versatility, these materials can be used in hospitals to print wound dressings for patients on demand. 

There are more potential applications for these environmentally-friendly materials. A clear example of this is food packaging. There are some antioxidants on the market that can be combined with plastics for food packaging [50,51]. Antioxidant packaging can be used to improve the condition and increase the shelf-life of packaged food. Due to the enhanced cell proliferation on antioxidant materials [16], these materials can be used for tissue culture applications or even for regenerative medicine. Due to the versatility of FFF, complex geometries can be prepared such as scaffolds. However, before this type of materials can be implanted into humans, the safety of lignin-based materials should be evaluated. It has been reported before that LIG-based materials are biocompatible [45] but more studies should be performed.
